# Sophisticated perspective-takers are distinctive: Neural idiosyncrasy of functional connectivity in the mentalizing network

**DOI:** 10.1016/j.isci.2024.111472

**Published:** 2024-11-23

**Authors:** Yu Zhang, Chao Ma, Haiming Li, Leonardo Assumpção, Yi Liu

**Affiliations:** 1 School of Psychology, Northeast Normal University, Changchun 130024, China; 2 Jilin Provincial Key Laboratory of Cognitive Neuroscience and Brain Development, Changchun 130024, China; 3 General and Experimental Psychology, Department of Psychology, Ludwig-Maximilians University, 80802 Munich, Germany

**Keywords:** Behavioral neuroscience, Cognitive neuroscience, Neuroscience, Social sciences

## Abstract

Naive perspective-takers often perceive the social world in a simplistic and uniform way, whereas sophisticated ones recognize the diversity and complexity of others’ minds. This commonly accepted distinction points to a possibility of greater inter-individual variability in mentalizing for sophisticated than naive perspective-takers, a difference previously overlooked in research. In the current study, participants were asked to watch a mentalizing-related movie and their neural responses, interpretations of the characters’ mental states, and eye-gaze trajectories were recorded. The results provide robust and converging evidence that the neural connectomic features within the mentalizing network, eye-gaze trajectories, and interpretations of others’ mental states exhibit greater inter-individual variability among sophisticated perspective-takers compared to naive ones, supporting that sophisticated perspective-takers are more distinctive while naive ones are more similar. These findings deepen our understanding of mentalizing by highlighting the idiosyncrasy and homogeneity of neural collaboration and behavioral manifestations across varying levels of perspective-taking sophistication.

## Highlights

• Sophisticated perspective-takers are distinctive and naive ones are similar (AnnaK effect)
• The AnnaK effects were shown on the connectomic features of the mentalizing network
• The eye-gaze trajectories and interpretations of others’ mind also showed AnnaK effect
• The findings deepen our understanding of mentalizing through inter-individual variability

## Graphical abstract

**Figure:**
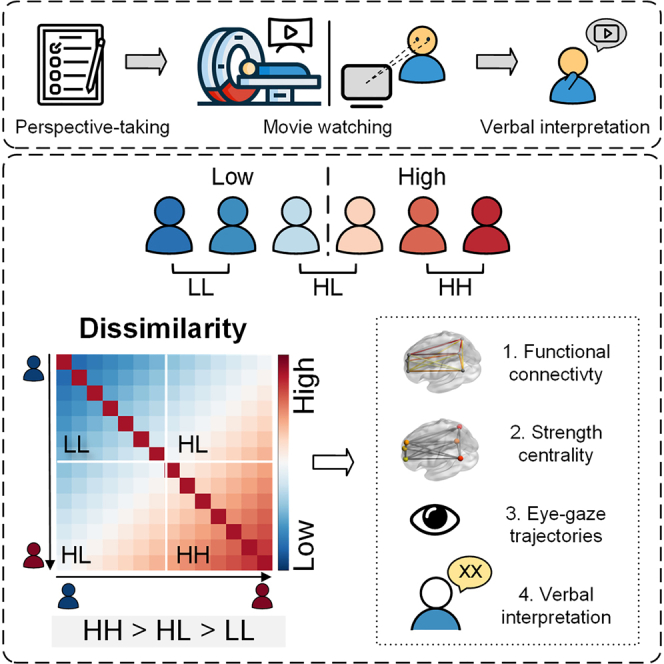

## Introduction

A group of friends decided to go out for lunch together, but Tom said he was not hungry and headed home alone. Such behavior invites various interpretations for different individuals. Those who are less experienced or naive in perspective-taking (PT) might consistently accept that Tom was not hungry, while for sophisticated perspective-takers, some might infer Tom was not in the mood and sought solitude, while others might assume he was saving money. This scenario raises an interesting question: Do sophisticated perspective-takers exhibit greater inter-individual variability in mentalizing compared to their naive counterparts? Exploring the inter-individual variability, especially the neural idiosyncrasy in mentalizing for individuals with different levels of PT is an intriguing yet largely unexplored area in both scientific research and everyday social interactions.

The mentalizing process is supported by the neural activities of a group of brain regions including the temporoparietal junction (TPJ), medial prefrontal cortex (MPFC), and the precuneus (PreC), widely known as mentalizing network (MTN).^1^^,^^2^^,^^3^ Most previous studies investigating differences between sophisticated and naive perspective-takers have primarily focused on the correlation between neural responses of the regions in the MTN and individuals’ level of PT. For example, individuals with higher levels of PT showed stronger activation of TPJ during observation of others’ pain,^4^ stronger activation of MPFC during observation of mentalizing animations^5^ and false-belief reasoning.^6^ But the PreC has been found to exhibit a negative correlation with PT scores in the false-belief task.^7^ These findings have merely illustrated the trend in which the degree of involvement of the MTN varies with individuals’ PT levels during mentalizing, while neglecting the differences in inter-individual variability between sophisticated and naive groups.

Inspired by the opening line of Leo Tolstoy’s novel “Anna Karenina”—"All happy families are alike; each unhappy family is unhappy in its own way”—researchers have developed the Anna Karenina (AnnaK) model. This model highlights the nuanced inter-individual variability within groups, suggesting that all high/low scorers are alike; each low/high scorer is different in his or her own way (for a comprehensive review, see Finn et al.^8^). The AnnaK model has garnered recent interest and has been empirically tested through calculating the inter-subject (dis)similarity of the neural responses and associating it with individuals’ behavioral indices. For instance, with the inter-subject correlations (ISCs) of the time series in the default mode network during video watching, Baek et al.^9^ demonstrated that individuals who are unpopular (low in-degree) in the social network are distinctive while the popular individuals (high in-degree) are similar. Similarly, Baek et al.^10^ showed that individuals with high levels of loneliness are distinctive while individuals with low levels of loneliness are similar. Focusing more on the process of social interpretation, Finn et al.^11^ showed that the inter-subject correlations of the neural responses in the MTN were greater for high-paranoia dyads than low-paranoia dyads, suggesting that individuals with low trait paranoia are distinctive, and those with high trait paranoia are similar when processing social narratives. These inter-individual neural (dis)similarities were all based on the time series of neural responses in single regions. Most recently, using the inter-subject dissimilarity of functional connectivity between regions as the index, Iyer et al.^12^ showed that individuals who tended to see the good in bad situations were distinctive, and those with more negative views were similar in the neural processing of others’ negative experiences. Taken together, it can be seen that a broad range of neural idiosyncrasy follows the AnnaK model. As the aforementioned example of Tom suggests, there would be greater variability in mentalizing for high scorers of PT (i.e., the sophisticated perspective-takers) than for low scorers (i.e., the naive perspective-takers). Nonetheless, evidence is still lacking as to whether sophisticated perspective-takers are distinctive and naive perspective-takers are similar in their neural responses during mentalizing.

**Figure 1:**
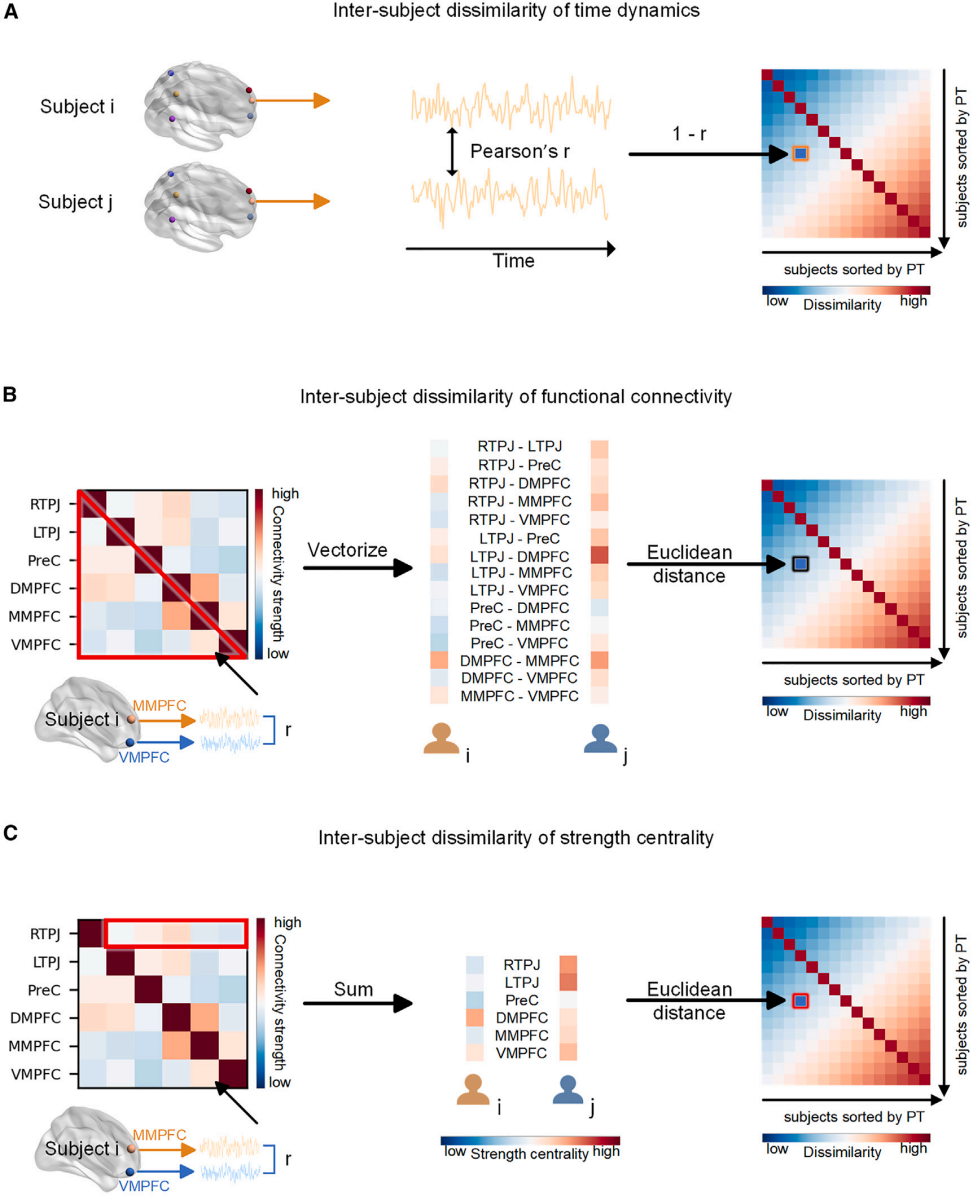
Calculation of inter-subject dissimilarity (A) Inter-subject dissimilarity of time dynamics for each region was calculated as 1 minus the correlation coefficient of the time series extracted from each region between pairs of participants. (B) For each participant, the functional connectivity matrix for the MTN was established. The inter-subject dissimilarity of the global functional connectivity was defined as the Euclidean distance between vectorized 15 connections between 6 regions for each dyad. (C) The strength centrality for each region was calculated as the sum value of its connectivity with the other five regions in the MTN. The inter-subject dissimilarity of the global strength centrality was represented by the Euclidean distance between the strength centrality of the 6 regions for each dyad. RTPJ, right temporoparietal junction; LTPJ, left temporoparietal junction; PreC, precuneus; DMPFC, dorsal medial prefrontal cortex; MMPFC, middle medial prefrontal cortex; VMPFC, ventral medial prefrontal cortex.

To address this issue, we categorized participants (fMRI experiment: *N* = 55; eye-movement experiment: *N* = 41) as either sophisticated (i.e., high PT) or naive (i.e., low PT) perspective-takers according to their PT scores (measured by the PT subscale of Interpersonal Reactivity Index (IRI) scale^13^). Neural responses of the MTN were recorded with functional magnetic resonance imaging (fMRI) while participants watched a silent video “Partly Cloudy”, which has been proven to effectively induce MTN activation.^14^^,^^15^^,^^16^ Given that the mentalizing process relies on both the specific function of each region^17^^,^^18^^,^^19^ and the functional connectivity profile of the MTN,^3^^,^^20^ the neural idiosyncrasy was indexed by the pairwise inter-subject dissimilarity of both the regional and connectomic features of the MTN. Specifically, our focus was on three indices: time dynamics of the neural responses of single regions, functional connectivity between regions, and the strength centrality (based on the functional connectivity) of regions within the MTN (Figure 1). The inter-subject dissimilarity of these three indices was compared between high-high (HH), high-low (HL), and low-low (LL) PT dyad groups to examine whether the neural idiosyncrasy during mentalizing supports the AnnaK model, positing that sophisticated perspective-takers are more distinctive, and naive perspective-takers are more similar. To assess the behavioral manifestation of the AnnaK effect, the eye-gaze trajectories during movie watching were recorded in an independent eye-movement experiment, as well as the verbal interpretations of others’ mental states. The inter-subject dissimilarities of these two behavioral indices were calculated.

## Results

### The involvement of MTN

Six regions within the MTN—specifically, dorsal MPFC (DMPFC), medial MPFC (MMPFC), ventral MPFC (VMPFC), PreC, left TPJ (LTPJ), and right TPJ (RTPJ)—were designated as regions of interest (ROIs) based on prior research.^16^ Subsequently, the time series data from these ROIs were extracted for further analyses (see STAR Methods for details).

In order to confirm the extensive involvement of the MTN during watching the mentalizing-related movie compared to rest, we conducted the temporal ISCs. These were computed as Pearson correlations between the time series data of each dyad across six regions within the MTN during video watching and resting states. This methodological approach assumes that voxels exhibiting high correlations in their time courses across subjects play a stereotyped functional role in processing the stimulus.^21^ Results showed that all six ROIs exhibited significantly higher ISCs during watching the mentalizing-related movie compared to rest (*ps* < 0.001, Table S1). Furthermore, we assessed functional connectivity using these six ROIs as seeds with all other regions in the brain (154 regions from the Schaefer2018 atlas,^22^ excluding regions in the default mode network that highly overlap with our ROIs). Results showed that during movie watching, these ROIs demonstrated stronger connectivity within the MTN than with regions outside the MTN (*t*(54) = 21.034, *p* < 0.001). These findings provide evidence that the six ROIs selected from prior research,^16^ constitute a coherent functional network and are robustly involved in mentalizing during video watching.

### The neural idiosyncrasy of MTN

In the fMRI experiment, a total of 55 participants were recruited and divided into a high PT group (i.e., sophisticated perspective takers; *N* = 27) and a low PT group (i.e., naive perspective takers; *N* = 28) based on the median score of the PT measurement.^13^ Each subject was paired with another subject, resulting in 1485 dyads classified as either HH, HL, or LL dyads based on each subject’s PT score.

Prior to conducting the formal analyses, we identified several variables that were either correlated with PT or showed significant differences between the high and low PT groups as potential confounding factors (e.g., personality, empathy; see Table S2). These variables were then included as covariates to control for their influence (see STAR Methods for details).

#### Time dynamics for single regions in MTN

Firstly, we investigated whether the neural idiosyncrasy of regional features, i.e., the time dynamics of neural responses for single regions of the MTN, conforms to the AnnaK model. For each region, the inter-subject dissimilarity was calculated as 1 minus the correlation coefficient of the time series for each dyad (Figure 1A and STAR Methods), and subjected to a linear mixed-effects (LME) model for comparisons between dyad groups (i.e., HH, HL, and LL), with false-discovery rate (FDR) correction for multiple comparisons.^9^^,^^10^ The results revealed that the inter-subject dissimilarity of the dynamic neural responses was comparable across dyad groups (*p*s > 0.05, Table S3), with the exception that the VMPFC exhibited smaller inter-subject dissimilarity for the HH dyads compared to the HL dyads (β = - 0.216, SE = 0.087, *p*_corrected_ = 0.008). However, neither the difference between the HH and LL dyads (β = - 0.256, SE = 0.142, *p*_corrected_ = 0.065) nor the difference between the HL and LL dyads (β = - 0.040, SE = 0.087, *p*_corrected_ = 0.609) were significant. When regarding the PT as a continuous variable, the correlation between the mean PT scores and the inter-subject dissimilarity of the dynamic neural responses were also not significant (*ps* > 0.1, Table S4). These results suggest that the neural idiosyncrasy of the time dynamics for single regions during mentalizing does not align with the AnnaK model for sophisticated and naive perspective-takers.

#### Functional connectivity between regions in MTN

**Figure 2:**
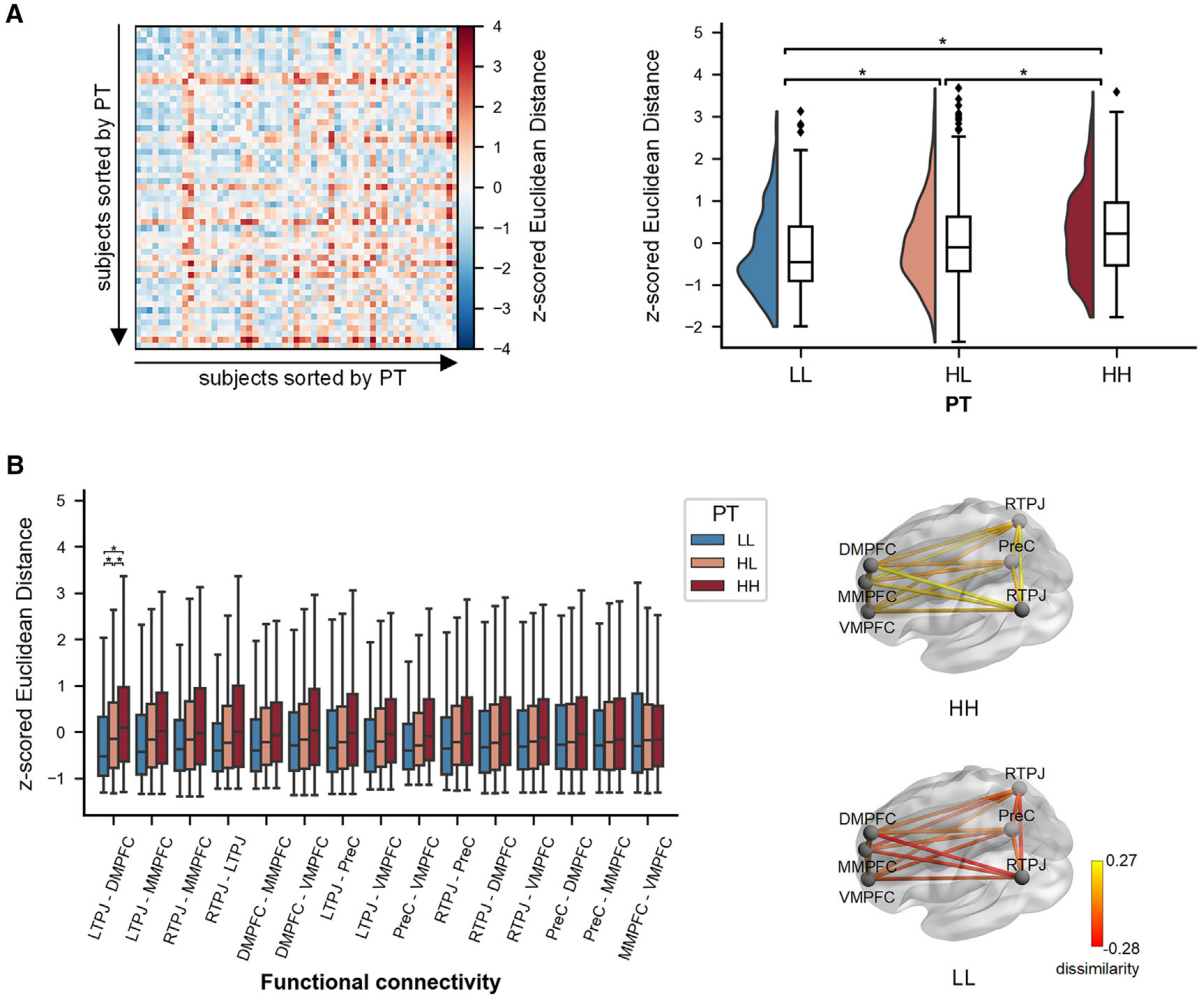
Results of inter-subject dissimilarity (i.e., Euclidean distance) in functional connectivity between regions (A) Left panel: inter-subject dissimilarity matrix of the functional connectivity within the mentalizing network; the rows and columns of the matrix are ordered by increasing perspective-taking (PT) scores. The colors stand for the standardized Euclidean distance of the global functional connectivity for each dyad. Right panel: inter-subject dissimilarity in three dyad groups (i.e., HH, HL, and LL). (B) Left panel: inter-subject dissimilarity in three dyad groups for single connection within the mentalizing network. Significance was shown after FDR corrections. Right panel: mean standardized inter-subject dissimilarity of connections for HH and LL dyad groups. The half-violin plots represent the distribution of data. Boxes represent the interquartile range; horizontal lines indicate the median and whiskers extend 1.5 times the interquartile range from the first and third quartiles. ∗*p* < 0.05. RTPJ, right temporoparietal junction; LTPJ, left temporoparietal junction; PreC, precuneus; DMPFC, dorsal medial prefrontal cortex; MMPFC, middle medial prefrontal cortex; VMPFC, ventral medial prefrontal cortex.

Moreover, we examined whether the neural idiosyncrasy of connectomic features, i.e., the functional connectivity between regions of the MTN, corresponds with the AnnaK model. First, we computed Pearson correlations between the time series of each pair of regions for each participant to construct the functional connectivity matrix of the MTN. Then, we calculated the Euclidean distance between the vectorized 15 connections across the six regions for each dyad to quantify the inter-subject dissimilarity of the functional connectivity profile (Figure 1B). Subsequently, an LME model was fitted to compare the inter-subject dissimilarity between dyad groups (i.e., HH, HL, and LL). The results showed the largest inter-subject dissimilarity for HH dyads compared to HL dyads (β = 0.289, SE = 0.152, *p*_corrected_ = 0.014) and LL dyads (β = 0.546, SE = 0.296, *p*_corrected_ = 0.014). The inter-subject dissimilarity for HL dyads was also larger than LL dyads (β = 0.257, SE = 0.152, *p*_corrected_ = 0.017) (Figure 2A). These findings demonstrate that individuals with high levels of PT exhibit greater inter-individual variability in functional connectivity within the MTN compared to individuals with low levels of PT. To validate our findings using both a median-split approach and treating PT as a continuous variable, we examined the correlation between the mean PT values of dyads and the inter-subject dissimilarity of global functional connectivity. The positive correlation (β = 0.229, *p* = 0.004) reaffirms that sophisticated perspective-takers are more distinctive and naive ones are more similar.

Subsequent analyses aimed to identify the connections within the MTN that contribute to the greater variability among sophisticated perspective-takers compared to naive perspective-takers. The paired Euclidean distance of each single connection was calculated and subjected to an LME model for group comparison, with FDR corrections for multiple comparisons across connections and comparisons. Results revealed that the connection between LTPJ and DMPFC showed significantly larger inter-subject dissimilarity for HH dyads compared to both LL dyads (β = 0.625, SE = 0.275, *p*_corrected_ = 0.030) and HL dyads (β = 0.300, SE = 0.143, *p*_corrected_ = 0.044). The differences between HL dyads and LL dyads were also significant (β = 0.325, SE = 0.143, *p*_corrected_ = 0.030). Notably, although the results of other connections did not reach significance, the trends were similar (Figure 2B and Table S5). This indicates that sophisticated perspective-takers demonstrate distinct functional connectivity profiles within the MTN, while naive individuals exhibit similarities, aligning with the AnnaK model.

In addition to inter-individual variability, the strength of each connection and the mean strength of all connections within the MTN were compared between the high and low PT groups. No significant group difference in the mean functional connectivity of the MTN (*t*(53) = - 0.895, *p* = 0.375) or in single connections was found (*p*s > 0.1, Table S6), suggesting that sophisticated and naive perspective-takers do not differ in the mean collaboration levels of the MTN.

#### Strength centrality for regions based on functional connectivity in MTN

**Figure 3:**
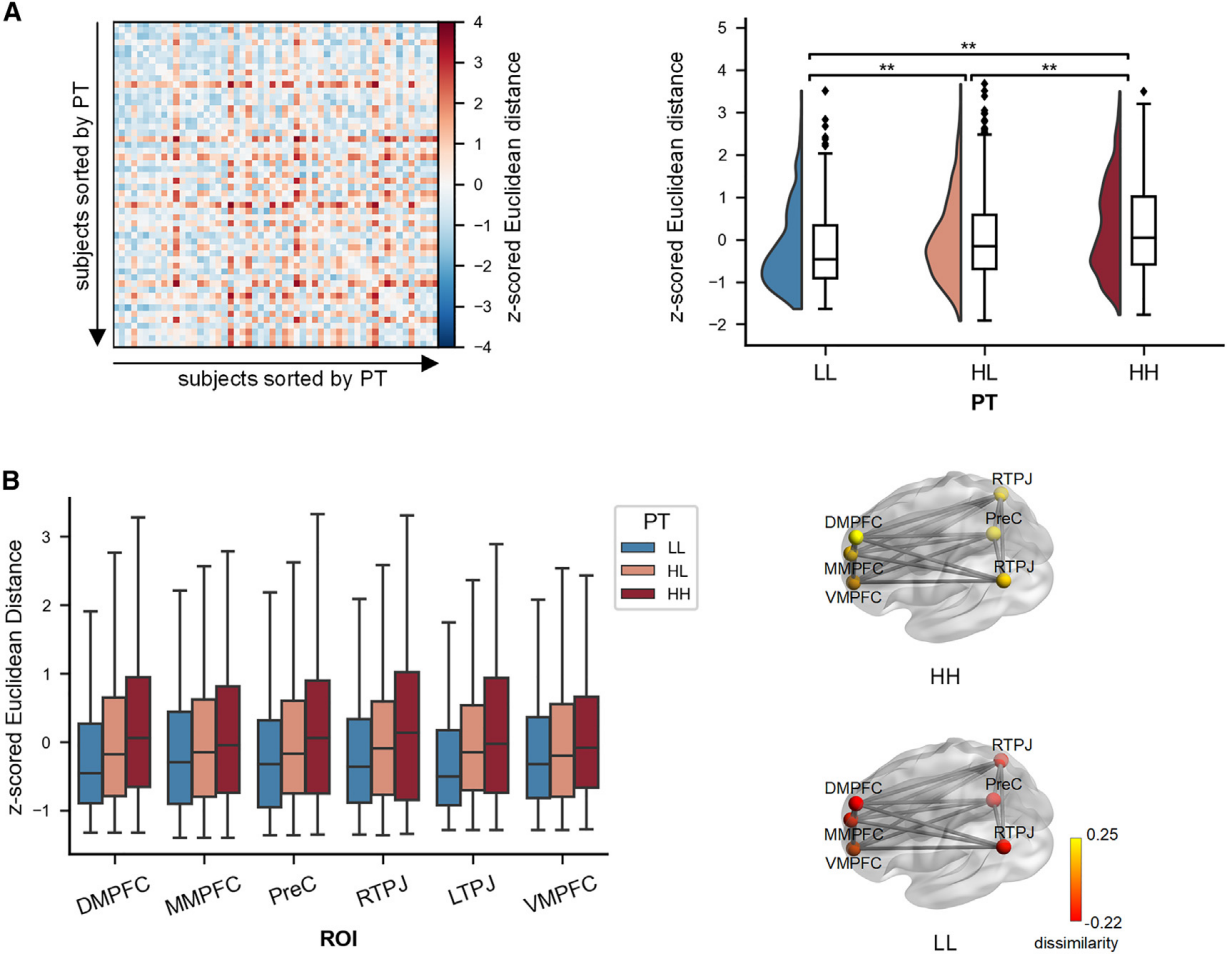
Results of inter-subject dissimilarity (i.e., Euclidean distance) in strength centrality (A) Left panel: inter-subject dissimilarity matrix of the global strength centrality within the mentalizing network; the rows and columns of the matrix are ordered by increasing perspective-taking (PT) scores. The colors stand for the standardized Euclidean distance of the global strength centrality for each dyad. Right panel: inter-subject dissimilarity in three dyad groups (i.e., HH, HL, and LL). (B) Left panel: inter-subject dissimilarity of strength centrality in three dyad groups for each region within the mentalizing network. Significance was shown after FDR corrections. Right panel: mean standardized inter-subject dissimilarity of regional strength centrality for HH and LL dyad groups. The half-violin plots represent the distribution of data. Boxes represent the interquartile range; horizontal lines indicate the median and whiskers extend 1.5 times the interquartile range from the first and third quartiles. ∗∗*p* < 0.01. RTPJ, right temporoparietal junction; LTPJ, left temporoparietal junction; PreC, precuneus; DMPFC, dorsal medial prefrontal cortex; MMPFC, middle medial prefrontal cortex; VMPFC, ventral medial prefrontal cortex.

Apart from the functional connectivity between regions, the strength centrality, describing the extent to which a single region is connected with other regions within the network,^23^^,^^24^ could also be used to demonstrate neural idiosyncrasy. Thus, based on the established functional connectivity matrix for the MTN, we calculated the strength centrality for each region as the sum value of its connectivity with the other five regions in the MTN. The Euclidean distance between the strength centrality of the six regions for each dyad was defined as the inter-subject dissimilarity of the strength centrality within the MTN (Figure 1C and STAR Methods). Subsequently, the inter-subject dissimilarity of the strength centrality was compared between HH, HL and LL dyad groups using the LME model. Results revealed that the inter-subject dissimilarity for HH dyads was larger compared to HL dyads (β = 0.268, SE = 0.138, *p*_corrected_ = 0.009), and to LL dyads (β = 0.520, SE = 0.262, *p*_corrected_ = 0.009). The inter-subject dissimilarity was also larger for HL dyads than LL dyads (β = 0.252, SE = 0.137, *p*_corrected_ = 0.009) (Figure 3A). Similarly, regarding PT as a continuous variable, the correlation between the mean PT scores and the inter-subject dissimilarity of global strength centrality was also significant (β = 0.186, *p* = 0.008). This implies that sophisticated perspective-takers exhibit greater variability in the strength centrality of the MTN compared to naive perspective-takers, which is in line with the AnnaK model.

To determine the strength centrality of which regions contributed to the larger variability for sophisticated versus naive perspective-takers, we calculated the paired Euclidean distance of strength centrality for each single region and subjected it to LME for group comparisons, with FDR corrections applied for multiple comparisons. The DMPFC, MMPFC, PreC, and RTPJ showed similar trends to that of the global strength centrality. That is, the inter-subject dissimilarity for HH dyads was larger than HL and LL dyads and the differences between the HL dyads and LL dyads were also marginally significant after FDR correction (*ps* < 0.1) (Figure 3B and Table S7).

Additionally, by aggregating inter-subject variability, we compared the group mean strength centrality for each region between the high PT and low PT groups. No significant differences were found (*p*s > 0.1, Table S8), indicating that the sophisticated and naive perspective-takers exhibited similar mean strength centrality of each node in MTN, unless considering the variability of the group.

### The behavioral idiosyncrasy of mentalizing

#### Eye-gaze trajectories

**Figure 4:**
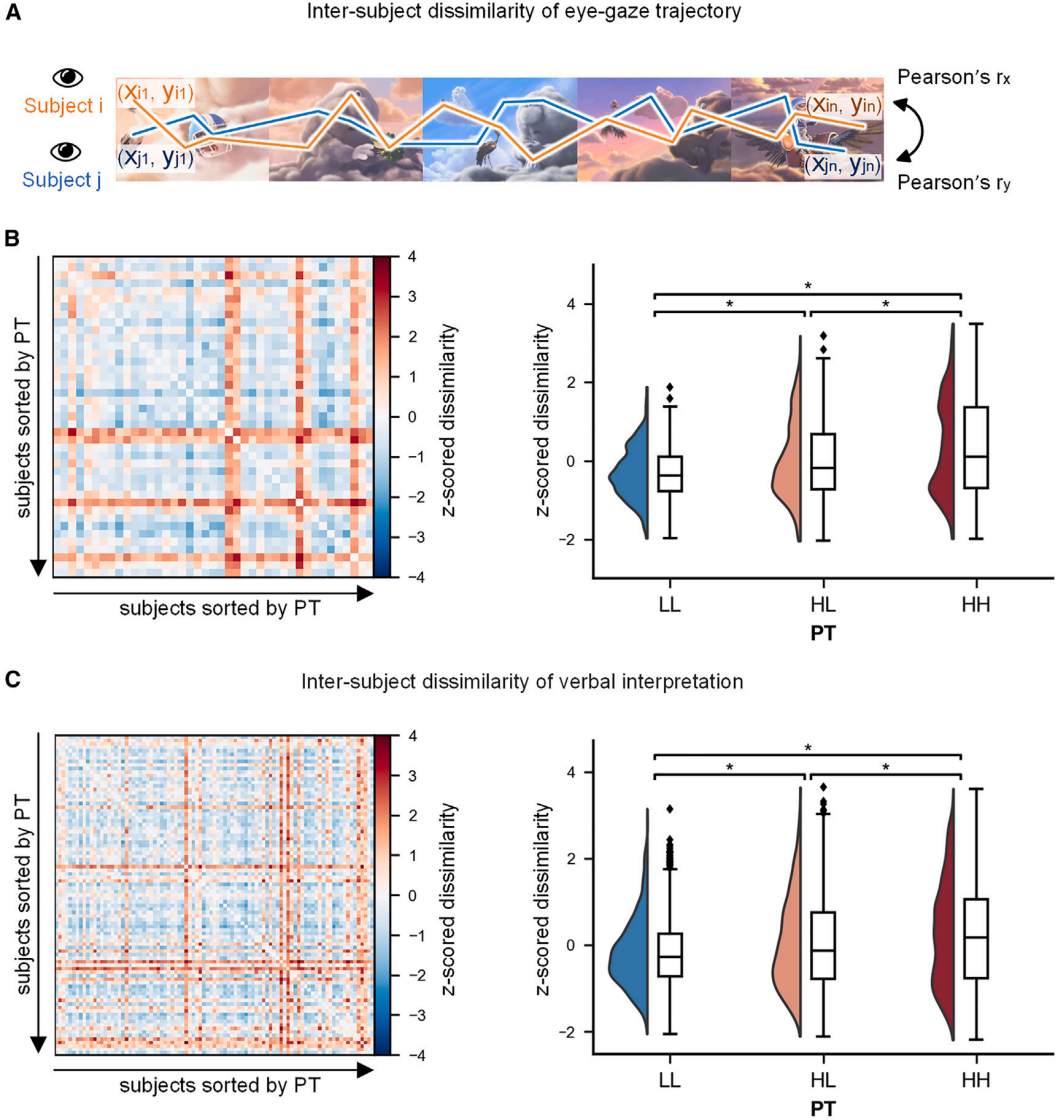
Calculation of inter-subject dissimilarity of eye-gaze trajectories and results for eye-gaze trajectories and verbal interpretation (A) Pearson’s correlation for gaze positions between pairs of participants in both the vertical and horizontal directions. The inter-subject dissimilarity was calculated as 1- (r_x_ + r_y_)/2. (B and C) Left panel: Inter-subject dissimilarity matrix of the eye-gaze trajectories and verbal interpretation; the rows and columns of the matrix are ordered by increasing PT scores. The colors in the left panels stand for the standardized dissimilarity of the eye-gaze trajectories and verbal interpretation for each dyad. Right panel: inter-subject dissimilarity in three dyad groups (i.e., HH, HL, and LL). The half-violin plots represent the distribution of data. Boxes represent the interquartile range; horizontal lines indicate the median and whiskers extend 1.5 times the interquartile range from the first and third quartiles. ∗*p* < 0.05.

Given that gazes on scenes can reflect individuals' mental processing of socially relevant information,^25^^,^^26^^,^^27^^,^^28^ we conducted an independent eye-movement experiment to provide a behavioral manifestation of the AnnaK effect for sophisticated and naive perspective-takers. Forty-one participants were divided into a high PT group (*N* = 20) and a low PT group (*N* = 21) (see STAR Methods for details). Participants’ eye-gaze trajectories were recorded while they watched the same movie used in the fMRI experiment. The inter-subject dissimilarity of the eye-gaze trajectories was calculated as 1 minus the averaged inter-subject Pearson correlations of the time series of the horizontal (x coordinates) and vertical (y coordinates) positions of gazes (i.e., 1− (r_x_ + r_y_)/2)^29^ (Figure 4A). An LME model was employed to compare the inter-subject dissimilarity of eye-gaze trajectories between dyad groups.

Results showed larger inter-subject dissimilarity for HH dyads compared to HL dyads (β = 0.463, SE = 0.231, *p*_corrected_ = 0.011) and compared to LL dyads (β = 0.878, SE = 0.460, *p*_corrected_ = 0.011). The difference was also larger for HL dyads than it was for LL dyads (β = 0.415, SE = 0.231, *p*_corrected_ = 0.011) (Figure 4B). These results demonstrate greater inter-individual variability in visual information gathering during mentalizing for sophisticated perspective-takers than naive ones, aligning with the AnnaK model and corroborating the fMRI results. However, when regarding the PT as a continuous variable, the correlation between the mean PT scores and the dissimilarity of the eye-gaze trajectories were not significant (β = 0.136, *p* = 0.297).

#### Verbal interpretation of others’ mental states

Given that participants’ verbal descriptions of a story can be coded to reflect their spontaneous mentalizing during watching salient movies,^30^ we used participants’ verbal interpretation of the characters’ mental state as an index to provide another behavioral manifestation of the AnnaK effect. The semantic similarity of all the mentalizing-related sentences in the verbal recollection was calculated^31^^,^^32^ for each dyad (see STAR Methods for details). The inter-subject dissimilarity of verbal interpretations (calculated as 1 minus the semantic similarity value) was analyzed using an LME model for group comparisons.

The results revealed greater inter-subject dissimilarity for HH dyads compared to HL dyads (β = 0.241, SE = 0.138, *p*_corrected_ = 0.013) and LL dyads (β = 0.488, SE = 0.274, *p*_corrected_ = 0.013). Inter-subject dissimilarity was also higher for HL dyads than for LL dyads (β = 0.247, SE = 0.137, *p*_corrected_ = 0.013) (Figure 4C). Similarly, inter-subject dissimilarity was positively correlated with the mean PT scores of each dyad when PT was considered as a continuous variable (β = 0.167, *p* = 0.020). These results demonstrate the AnnaK effect, i.e., sophisticated perspective-takers exhibited greater inter-individual variability in interpretations of others’ mental states compared to naive ones, consistent with the fMRI results.

### Associations between neural and behavioral idiosyncrasies

We have identified converging evidence of AnnaK effects on neural indices (i.e., functional connectivity and strength centrality) and behavioral indices (i.e., eye-trajectory and verbal interpretation). Further, we used Pearson correlations to explore whether dyads exhibiting more distinct functional connectomic features in the MTN also showed greater variability in their interpretations of the movie, assessed using Mantel tests (10,000 permutations). The results showed a positive trend in the correlation between inter-subject dissimilarity in verbal interpretations and global functional connectivity (*r*(50) = 0.128, *p* = 0.086). When focusing on the DMPFC-LTPJ connection that exhibited a significant AnnaK effect, the positive association reached significance (*r*(50) = 0.198, *p* = 0.016). This association suggests that inter-individual variability in the functional connectivity of the MTN during mentalizing may contribute to the diversity observed in how individuals interpret others’ mental states. However, the association between verbal interpretations and global strength centrality was not significant *(r*(49) = 0.071, *p* = 0.196).

Additionally, in the eye-movement experiment, a positive and marginally significant correlation was found between the inter-subject dissimilarity of verbal interpretation and that of eye-gaze trajectories (*r*(37) = 0.216, *p* = 0.067). This implies that variability in visual information processing during mentalizing is associated with diverse interpretations of others’ mental states.

### Controlling analyses

To confirm that our findings are robust and not derived from other confounding variables, we conducted a series of controlling analyses.

First, to determine whether the AnnaK effects on neural idiosyncrasy during mentalizing were specific to the MTN, we conducted parallel analyses on the physical pain network (Table S9), which was selected as a control network for the MTN in previous research.^16^^,^^33^ There were no differences between dyad groups (*ps* > 0.1, Table S10) for the two neural idiosyncrasy indices (i.e., inter-subject dissimilarity of the global functional connectivity and strength centrality), with the exception that the inter-subject dissimilarity of strength centrality exhibited larger inter-subject dissimilarity in the HL dyads compared to the LL dyads (*p*_corrected_ = 0.035). However, neither the difference between the HH and LL dyads (*p*_corrected_ = 0.132) nor the difference between the HH and HL dyads (*p*_corrected_ = 0.460) were significant. Moreover, we observed significant 3 (dyad group: HH vs. HL vs. LL) × 2 (network: MTN vs. Pain) interactions for global functional connectivity (*F*(2, 5824.87) = 22.026, *p* < 0.001) and strength centrality (*F*(2, 5610.75) = 13.233, *p* < 0.001). These results suggest that the greater variability in the functional connectomic features among sophisticated compared to naive perspective-takers is specific to the MTN and does not extend to networks unrelated to mentalizing.

Second, to further validate that our neural findings are linked to mentalizing, we segmented the movie into one mentalizing-unrelated segment (background part: 1–50 TRs) and two mentalizing-related segments (51–110 TRs and 111–151 TRs) based on content (see STAR Methods for details). If the AnnaK effects are specific to mentalizing, we would expect these effects to be evident in the last two mentalizing-related segments but not in the first mentalizing-unrelated segment. As anticipated, the inter-subject dissimilarity of global functional connectivity and strength centrality did not differ significantly between dyad groups in the background segment (*ps* > 0.1, Table S11). While in the mentalizing-related segments, the inter-subject dissimilarity for HH dyads was larger than the HL and LL dyads, and the inter-subject dissimilarity for HL dyads is also larger or marginally larger than LL dyads after FDR corrections across all comparisons and all segments (*ps* < 0.1, Table S11). These findings confirm that the AnnaK effects of neural idiosyncrasy in the MTN are specific to the mentalizing process.

Lastly, to verify that the AnnaK effect pertains to social information processing rather than general intrinsic neural activity, we conducted parallel analyses on neural activity in the MTN during rest. However, resting-state neural activity did not exhibit similar AnnaK effects observed during movie watching. The results showed that the inter-subject dissimilarity of the global functional connectivity and strength centrality was comparable across dyad groups (ps > 0.1, Table S12). We also included the inter-subject dissimilarity of the neural indices from the resting state in the LME as covariates and found that the AnnaK effects remained consistent (Table S13). Furthermore, the Pearson correlations between the neural indices of the resting state and verbal interpretation were not significant (functional connectivity: *r*(48) = −0.061, *p* = 0.264; strength centrality: *r*(48) = −0.077, *p* = 0.196; Mantel tests with 10,000 permutations). Therefore, we conclude that the neural idiosyncrasy of sophisticated perspective-takers does not stem from their intrinsic neural activity.

## Discussion

Do sophisticated perspective-takers exhibit greater inter-subject variability during mentalizing, while naive ones are more homogeneous? Our results demonstrated that the functional connectomic features of the MTN, alongside the eye-gaze trajectory and verbal interpretations on others’ mental states support the AnnaK model. That is, during mentalizing, all sophisticated perspective-takers are more distinctive in neural collaboration, visual information gathering, and interpretations on others’ mental states, whereas naive ones are more similar. These findings provide the first behavioral and neural evidence supporting the intuitive understanding that naive perspective-takers perceive the social world more simply and uniformly, while sophisticated perspective-takers see the inner worlds of others as rich tapestries, fostering diverse and distinctive interpretations among observers.

It is notable that the AnnaK effect does not encompass the time dynamics of the neural responses of isolated regions. In contrast, it was robustly shown in indices based on the functional connectivity in MTN, i.e., functional connectivity between regions and the strength centrality of the regions. From the perspective of the brain network, functional connectivity describes the collaboration of regions and serves as a person-specific “fingerprint”. It captured significant inter-subject variability in executive control,^34^ long-term memory,^35^ creativity,^36^ trait openness,^37^ empathy,^38^ interpersonal closeness,^39^ affect reactions,^12^ and personal identity.^40^ Moreover, the functionally heterogeneous regions of the MTN are known to be interconnected with highly structured fiber tracts and a unique hierarchical functional architecture that supports mentalizing.^3^ However, previous research focusing on the brain activities of single regions and their correlation with individuals’ PT^4^^,^^5^^,^^6^^,^^7^ overlooked the neural idiosyncrasy of functional connectivity. Our findings demonstrate that functional connectomic features in the MTN serve as acute indices signaling the inter-subject variability during mentalizing for sophisticated and naive perspective-takers.

Throughout brain development, functional connectivity within the MTN is observed to strengthen alongside the development of mentalizing abilities in children aged 3–12 years.^16^ Thus, the comparable strength centrality and functional connectivity between sophisticated and naive perspective-takers when aggregating individual differences suggests indistinguishable functional maturity of the MTN across these groups. That means, naive perspective-takers also possess a highly functionally specialized social brain that allows them to engage in social interactions. Yet, the functioning patterns of their MTN may be relatively less flexible, resulting in reduced variability for naive perspective-takers compared to sophisticated ones. This suggests that the distinctiveness observed in sophisticated perspective-takers and the homogeneity seen in naive individuals are more likely driven by the collaborative dynamics within the MTN rather than solely by its maturity. In addition, no analogous effect was observed in the physical pain network or in the resting state activity in the MTN, implying that the AnnaK effect distinguishing sophisticated and naive perspective-takers is specific to the collaboration of MTN rather than being a widespread phenomenon across the brain, and is specific to mentalizing processing rather than intrinsic connectivity.

In the MTN, the PreC is known to be sensitive to others’ visual and psychological perspectives,^41^^,^^42^^,^^43^^,^^44^ while the TPJ is responsible for inferring others’ temporary mental states, such as goals, intentions, and desires.^45^ The ventral and medial regions of MPFC are critical for self-reflection^46^ and self-other distinctions,^47^^,^^48^ whereas the dorsal part is involved in processing information of others^2^ and inferring others’ traits and dispositions.^1^^,^^45^ Therefore, the considerable variability in the neural functioning of the MTN among sophisticated perspective-takers may indicate that, during mentalizing, they adopt diverse perspectives, seamlessly switch between their own viewpoint and that of others, and effectively integrate both self-related and other-related information to generate varied inferences about others’ goals, intentions, and desires. However, the precise cognitive mechanisms underlying this variability in MTN collaboration warrant further investigation in future studies.

Furthermore, the idiosyncrasy of eye-gaze trajectory and verbal interpretations about others’ mental states also exhibited similar AnnaK effects. Eye-gaze patterns are recognized as a sensitive indicator of one’s mental processing of social stimuli, such as assessment of others’ social status^25^ and understanding of false beliefs.^26^ In particular, the inter-subject similarity in dynamic eye-gaze trajectories has been documented to accurately reflect the perspectives that participants adopt.^27^^,^^28^ In our findings, the varied eye-gaze trajectories of sophisticated perspective-takers may reflect their engagement in gathering diverse information and dynamically switching perspectives during viewing, which contributes to variability in mentalizing. Moreover, individuals’ verbal interpretations of others’ mental states showed greater inter-individual variability among sophisticated perspective-takers compared to naive ones. This behavioral evidence provides a clearer understanding of the AnnaK effect in mentalizing: sophisticated perspective-takers may interpret others’ mental states more diversely, whereas naive individuals tend to have a relatively uniform understanding. These converging evidence from neural and behavioral indices strengthen the validity of the AnnaK effect in mentalizing across different levels of PT. Importantly, individuals who exhibit greater similarity in the functioning of the MTN are also likely to provide more similar interpretations of others’ mental states. This association between neural and behavioral demonstrations of the AnnaK effects suggests that inter-individual variability in the functioning of the MTN may underlie the diversity of interpretations of others’ mental states in the social world.

Taken together, our findings enrich our understanding of social interactions by highlighting the idiosyncratic neural collaboration within the MTN among sophisticated perspective-takers. Conversely, for naive perspective-takers, the social world might seem simpler due to their homogeneous mentalizing processes.

### Limitations of the study

It is imperative to acknowledge some limitations inherent in the current study. First, the video we used to induce mentalizing was somewhat explicit, allowing naive perspective-takers to comprehend it easily, even though its silent nature introduced a degree of ambiguity in certain scenes. This ambiguity permitted inter-individual variability among sophisticated perspective-takers. It is conceivable that in situations that are unambiguous yet obscure (e.g., on Valentine’s Day, a girl said to her boyfriend, “The flowers that girl is holding look beautiful.”), the inter-individual variability among sophisticated perspective-takers might decrease due to their accurate interpretation of the implied meanings, whereas it might increase among naive ones due to confusion and speculative interpretations. Thus, the impact of ambiguity and obscurity of the situations warrants further exploration to provide precise contextual constraints on the manifestation of the AnnaK effect in mentalizing. Second, our classification of sophisticated and naive perspective-takers was based on the PT subscale of the IRI,^13^ reflecting a combination of the tendency and ability of the respondent to adopt the perspectives of other people. In real life scenarios, some individuals may possess the high ability to adopt others’ perspectives but lack the inclination to do so, thereby acting as sophisticated perspective-takers only when deemed necessary. Differentiating between the tendency and ability to engage in PT could provide deeper insights into the large inter-individual variability in mentalizing observed among sophisticated perspective-takers. Lastly, while we used the pain network as a control to demonstrate that the AnnaK effect is specific to the MTN, this does not imply that the mentalizing process is exclusively reliant on the MTN. Future research should investigate the interplay among different networks to offer a comprehensive view of the inter-individual variability in mentalizing.

## Resource availability

### Lead contact

Further information and requests for resources should be directed to and will be fulfilled by the lead contact, Yi Liu (liuy930@nenu.edu.cn).

### Materials availability

This study did not generate new unique reagents.

### Data and code availability

• The behavioral data and processed fMRI data have been deposited on GitHub and are publicly available as of the date of publication. Accession numbers are listed in the key resources table.
• Original codes have been deposited on GitHub and are publicly available as of the date of publication. Accession numbers are listed in the key resources table.
• Any additional information required to reanalyze the data reported in this article is available from the lead contact on request.

## Acknowledgments

This study was supported by the 10.13039/501100001809National Natural Science Foundation of China (NO.31800957).

## Author contributions

Conceptualization, Y.L.; Methodology, Y.L.; Software, H.L.; Formal analysis, Y.Z.; Investigation Y.Z. and C.M.; Writing – Original Draft, Y.Z. and Y.L.; Writing – Review and Editing Y.Z., L.A., and Y.L.; Visualization, Y.Z.; Supervision, Y.L.

## Declaration of interests

The authors declare no competing interests.

## STAR★Methods

### Key resources table

**Table:** 

REAGENT or RESOURCE	SOURCE	IDENTIFIER
**Deposited data**		

Behavioral data	This paper	https://github.com/yu47726/Sophisticated-perspective-takers-are-distinctive.git
Processed data	This paper	https://github.com/yu47726/Sophisticated-perspective-takers-are-distinctive.git
Code	This paper	https://github.com/yu47726/Sophisticated-perspective-takers-are-distinctive.git

**Software and algorithms**		

MATLAB	Mathworks	https://de.mathworks.com/; RRID: SCR_001622
SPM12	The Wellcome Center for Human Neuroimaging	https://www.fil.ion.ucl.ac.uk/spm; RRID: SCR_007037
R 4.3.2	R Core Team	https://www.r-project.org/; RRID: SCR_001905
R package “lme4”	(Bates et al., 2015)^49^	https://cran.r-project.org/web/packages/lme4/index.html; RRID: SCR_015654
R package “lmerTest”	(Kuznetsova et al., 2017)^50^	https://cran.r-project.org/web/packages/lmerTest/index.html; RRID: SCR_015656
Text2Vec	Open-source application for text analysis	https://github.com/shibing624/text2vec

### Experimental model and study participant details

In the fMRI experiment, 55 college students participated (31 females; mean = 20.8 years, SD = 1.64 years), after excluding one participant due to equipment malfunction. 52 out of 55 participants were also engaged in the resting-state scanning. The subjective PT levels of participants were assessed using the PT subscale of the IRI,^13^ which consists of 7 items (e.g., “sometimes try to understand my friends better by imagining how things look from their perspective.”) with a maximum total score of 28. Based on the median score, participants were categorized into a high PT group (PT score >19; *N* = 27) and a low PT group (PT score ≤ 19; *N* = 28) (see Figure S1A). Each participant was paired with another, forming 1485 dyads classified as either HH, HL, or LL based on their respective PT group. Gender (*χ*^2^ = 0.439, *p* = 0.508) and age (*t*(53) = 0.915, *p* = 0.365) were matched between the high and low PT groups.

In the separate eye-movement experiment, 41 college students participated (21 females; mean = 20.4 years, SD = 1.36 years), after excluding two participants due to equipment malfunction. Participants were divided into high (PT score >18; *N* = 20) and low PT (PT score ≤ 18; *N* = 21) groups using a median split (Figure S1B), resulting in 820 dyads labeled as HH, HL, or LL. Two groups were matched in gender (*χ*^2^ = 0.223, *p* = 0.636) and age (*t*(39) = −0.384, *p* = 0.703).

All participants were of Asian descent, right-handed and had normal or corrected-to-normal vision. Informed consent was obtained prior to participation. The study was approved by the ethics committee of Northeast Normal University (No.2023029).

For each subject, we calculated the average dissimilarity of each index (i.e., functional connectivity, strength centrality, eye-trajectory and verbal interpretation) with other subjects and computed the z-scores across all subjects. Subjects whose average inter-subject dissimilarity with others was more than three standard deviations from the mean were excluded from further analyses. For the strength centrality index, one subject was excluded (z = 3.11). For the verbal interpretation index, one outlier was excluded (z = 3.45), and another four subjects were excluded because no mentalizing-related sentences were recognized in their verbal interpretations.

### Method details

#### Stimulus and procedure

In the fMRI experiment, the participants were asked to watch the silent version of “Partly Cloud” (https://www.pixar.com/partly-cloudy#partly-cloudy-1), an animated movie that has been used and validated for effectively inducing neural responses associated with mentalizing.^14^^,^^15^^,^^16^ The movie was silent and there was no explicit task during scanning, participants were instructed to watch and try to understand the story. They were informed that they would watch the movie again after scanning and recall what they thought about the story during scanning. Before the movie-watching scan, 52 out of 55 participants underwent a resting-state fMRI scan (180 TR), during which they were told to open their eyes and not think about anything.

In the eye-movement experiment, participants watched the same video while their eye trajectories were recorded. Similarly, they were asked to recall what they thought about the story after the eye-movement recording.

Since fMRI scanning did not allow for concurrent speech recording during scanning and the eye-movement recording required strict control of head movement, we asked participants to watch the movie again (at double speed, approximately 2.5 min) after the fMRI and eye-movement sessions. During this second viewing, they were instructed to concurrently recall the interpretations they had generated during the fMRI scan and eye-movement recording. These verbal reports served as the behavioral index for interpreting others’ mental states.

#### fMRI data acquisition

Brain images were acquired using a 3.0 T UIH uMR790 scanner with a 32-channel head coil. Functional images were recorded using an echo-planar sequence (64 × 64 × 36 matrix with 3.59 × 3.59 × 3.50 mm^3^ spatial resolution, repetition time = 2000 ms, echo time = 35 ms, flip angle = 90°, field of view = 23 × 23 cm). A black screen was included at the beginning (with duration = 8 s) to allow the BOLD signal to stabilize. A high-resolution T1-weighted structural image (256 × 256 × 160 matrix with 1.0 × 1.0 × 1.0 mm^3^ spatial resolution, repetition time = 7.9 ms, echo time = 3.1 ms, flip angle = 9°) was acquired before the functional scan.

#### Eye-movement recording

Eye-gaze trajectories were recorded using an Eyelink 1000 Plus desktop eye tracker, with a sampling rate of 1,000 Hz. The displayed video frame measured 1280 × 790 pixels and ran at 24 frames per second. It was presented on a 19-inch Liquid Crystal Display with a resolution of 1280 × 1024 and a refresh rate of 60 Hz. The eyes of the participant were about 60 cm from the center of the screen. A nine-point calibration and validation procedure were conducted prior to the experiment.

### Quantification and statistical analysis

#### fMRI data preprocessing

Functional images were preprocessed using SPM12 software (the Wellcome Trust Center for Neuroimaging, London, UK). The first four functional images were discarded. Standard preprocessing was applied, including correction for slice timing and head motion. Six movement parameters (translation; x, y, z, and rotation; pitch, roll, yaw) were extracted for further analysis in the statistical model. The anatomical image was co-registered with the mean realigned functional image and then normalized to the standard Montreal Neurological Institute (MNI) template. The functional images were resampled to 2 × 2 × 2 mm^3^ voxels, normalized to the MNI space using the parameters of anatomical normalization, and then spatially smoothed using an isotropic of 6 mm full-width half-maximum (FWHM) Gaussian kernel. Then, a general linear model was built for each subject to regress out head motion effects (6 corresponding rotation and translation parameters), with implicit high-pass filtering of 1/128 Hz. After model estimation, the residual images were saved for further analysis.

#### ROI definitions

The ROIs in the MTN were defined as 10mm spheres around the peak coordinates of RTPJ (x/y/z = 48–60 30), LTPJ (x/y/z = -48 -62 30), PreC (x/y/z = 0 –54 34), DMPFC (x/y/z = −6 54 36), MMPFC (x/y/z = −4 58 16), and VMPFC (x/y/z = −4 56 -16) reported in previous publication,^16^ in which the brain activity of the MTN was also induced during watching the “Partly Cloudy” movie. The “Pain Network” was also selected as a control network as ref. 16 for the current analyses (Table S9). Preprocessed time series data (151 TR) were extracted using MarsBaR (http://marsbar.sourceforge.net) from each ROI for each participant.

#### Calculations of neural inter-subject dissimilarity

##### Time dynamics for single regions

For each ROI, the Pearson correlation coefficients for the time series between pairs of participants were calculated (see Figure 1A). The inter-subject dissimilarity was defined as 1 minus the correlation coefficient, in accordance with previous studies.^51^^,^^52^^,^^53^ Note that we here use a dissimilarity measure instead of similarity for ease of interpretation (i.e., to reflect inter-individual variability).

##### Functional connectivity between regions

The pairwise Pearson correlation between the time series of each two regions within the MTN was defined as the functional connectivity of the MTN. This functional connectivity matrix for each subject was established using Fisher z-transformations of the correlation coefficients (see Figure 1B).

For the global functional connectivity profile of the MTN, the off-diagonal half of the functional connectivity matrix (15 connections between 6 regions) was flattened into a vector for each participant. Then, the Euclidean distance between the vectorized functional connectivity within the MTN was defined as the inter-subject dissimilarity of the global functional connectivity in the MTN (see Figure 1B), consistent with prior work.^12^ As for each connection, the inter-subject dissimilarity was defined as the pairwise Euclidean distance (i.e., the absolute value of numerical differences) for each dyad. These processes were similarly applied to the pain network for controlling analyses.

##### Strength centrality for regions based on functional connectivity

Regional strength centrality has been defined as the sum of the weights of the edges to which a region is connected within a network.^23^^,^^24^ In the current study, the sum of the functional connectivity values (Fisher z-transformed correlation coefficients) of one region with all other regions within the MTN was defined as the strength centrality for this region. The inter-subject dissimilarity of this regional strength centrality was calculated as the pairwise Euclidean distances (i.e., the absolute value of numerical differences) of the strength centrality for each dyad.

Moreover, for the whole MTN, Euclidean distances between the vectorized strength centrality of all six regions for each dyad were calculated to quantify the inter-subject dissimilarity of global strength centrality, emphasizing the relative centrality of all the regions within the MTN (see Figure 1C). These processes were similarly applied to the pain network for controlling analyses.

#### Calculations of inter-subject dissimilarity on eye-gaze trajectories

The eye-gaze trajectory defined as the time series of gaze positions on the screen, was exported on a frame-by-frame basis (7203 data points in total). Firstly, missing gaze data with gaps shorter than 75 ms were interpolated using linear methods, while gaps exceeding 75 ms (equivalent to 2 frames in this study) were identified as eye blinks.^54^ Subsequently, data points falling outside the video frame and those identified as eye blinks were eliminated. The analysis focused on the intersecting datasets for each dyad, ensuring assessments of inter-subject dissimilarity were based on identical time frames.

To assess the inter-subject dissimilarity, we first computed the Pearson’s correlations for gaze positions in both the horizontal (x coordinates) and vertical (y coordinates) directions for each dyad. The inter-subject dissimilarity was then defined as 1 minus the average of the inter-subject correlations for gaze positions in these two directions,^29^ i.e., inter-subject dissimilarity = 1- (r_x_ + r_y_)/2 (see Figure 4A).

#### Calculations of inter-subject dissimilarity on verbal interpretation about others’ mental states and its association with other indices

The recordings of the oral recall of participants’ interpretation of the story (*N* = 91, combining the participants from the fMRI and eye-movement experiments) were transcribed into text, with interjections (e.g., “um” or “uh”) and repeated words removed. To identify sentences related to mentalizing, three independent raters blind to the participants’ PT scores labeled each sentence as either mentalizing-related (descriptions of one’s internal state such as emotion, goal/intention, or belief) or not.^30^ Sentences labeled as mentalizing-related by at least two of the three raters were selected to calculate the inter-subject dissimilarity of verbal interpretations of others’ mental states in the story. The mean number of words within these mentalizing-related sentences was 73.165 (SD = 33.452) and matched between the high and low PT groups (*t*(89) = 0.160, *p* = 0.874).

The text from each participant, composed by concatenating their mentalizing-related sentences, was embedded into a 768-dimensional vector space using the Sentence-BERT (SBERT) model,^55^ implemented through the Python package Text2Vec (https://github.com/shibing624/text2vec). Unlike the basic BERT model, which generates word-level embeddings, SBERT creates document-level embeddings, producing a fixed-size 768-dimensional vector for an entire document that effectively captures its overall meaning. The cosine similarity values between each pair of these vectors were used to measure the semantic similarity between participants’ verbal interpretations of others’ mental states. The inter-subject dissimilarity of verbal interpretations was defined as 1 minus the similarity value.

Further, we tested the association between the inter-subject dissimilarity of verbal interpretation and that of neural indices (i.e., functional connectivity and strength centrality), as well as eye-gaze trajectory, using Pearson correlations with Mantel tests (*n* = 10,000 permutations).

#### Identifying potential confounding variables

To ensure that our findings were specifically related to PT and not derived from other variables, including gender, age, Big-Five personality traits^56^ (neuroticism, extraversion, openness, agreeableness, and conscientiousness), and other subscales measured by the IRI^13^ (i.e., fantasy, empathy, and personal distress). We assessed whether these variables correlated with PT scores or differed significantly between high and low PT groups in each experiment. In the fMRI experiment, fantasy and empathy showed correlations with PT, while in the eye-movement experiment, neuroticism, extraversion, agreeableness, and conscientiousness were correlated with PT. All participants in the two experiments used for the verbal interpretation analyses demonstrated correlations between PT and empathy, extraversion and agreeableness (see Table S2 for details). To exclude the potential influence of these confounding variables, participants were categorized into high and low groups based on their scores for these variables using a median-split approach, similar to PT categorization. The binarized values of these variables were included as covariates in the subsequent dyad-level analyses. Additionally, in later correlation analyses where PT was treated as a continuous variable, these variables were also included as covariates, using the mean values of the two dyad members for each variable.

#### Comparisons of the inter-subject dissimilarity between HH, HL and LL dyads

We employed LME models to compare inter-subject dissimilarity measures across HH, HL and LL dyads, an approach previously applied in studies comparing dyad-level data.^9^^,^^10^ This method accounts for the non-independence of data arising from repeated observations of each participant, who contributes to multiple dyads. In alignment with earlier recommendations, we ‘doubled’ the data to allow fully crossed random effects.^57^ Prior to statistical analysis, we adjusted the degrees of freedom to N - k, where N is the number of unique observations, and k is the number of fixed effects within the model (in our case, *N* = 1,485, k = 1). Linear mixed-effects models were constructed employing the lme4^49^ and lmerTest^50^ packages in R, with the inter-subject dissimilarity index as the dependent variable, dyad-level of PT groups (i.e., HH, HL, and LL) as the independent variable, dyad-level of groups for confounding variables (e.g., fantasy and empathy) as covariates, and included random intercepts for each individual within a dyad (i.e., “Participant 1” and “Participant 2”). Subsequent contrasts among the HH, HL and LL dyads were executed using the emmeans package in R. All variables were standardized, and the regression coefficients (β) were obtained as outputs. These analyses encompassed all inter-subject dissimilarity indices (i.e., time dynamics, functional connectivity, strength centrality, eye-trajectory and verbal interpretation). FDR corrections were applied to all *p*-values to account for multiple comparisons both between groups and across multiple dependent variables (e.g., 6 ROIs for time dynamics and strength centrality, and 15 connections for the functional connectivity), maintaining a significance threshold at *p* < 0.05.

#### Relating the neural and behavioral inter-subject dissimilarity with non-binarized PT scores

To validate our findings using both a median-split approach (binarizing high and low PT groups) and treating PT as a continuous variable, we examined the correlation between the mean PT values of dyads and the neural and behavioral idiosyncrasy indices (i.e., time dynamics of single regions, global functional connectivity, and strength centrality, eye-trajectory and verbal interpretation) using LME models.^9^^,^^10^ In these LME models, the inter-subject dissimilarities served as dependent variables, the mean PT scores of dyads were treated as the independent variable, and the mean values of confounding variables as covariates, with random intercepts for each individual within a dyad (e.g., “Participant 1” and “Participant 2”). Since the inter-subject dissimilarity of time dynamics was calculated for 6 ROIs respectively, FDR corrections at *p* < 0.05 were applied to adjust for multiple comparisons.

#### Relating the neural idiosyncrasies with behavioral idiosyncrasies

To associate neural idiosyncrasies with behavioral idiosyncrasies, we examined the correlations between neural and behavioral indices. To exclude the potential influence of the confounding variables, we first regressed out the mean values of these variables from each index using linear regression, and the residuals were then used for further correlation analyses.^12^ For instance, in the fMRI experiment, fantasy and empathy were identified as confounding variables due to their correlations with PT. A linear regression model was constructed with the inter-subject dissimilarity of global functional connectivity as the dependent variable and fantasy and empathy as independent variables. The residuals were subsequently used to conduct Pearson correlations to explore the relationships between neural and behavioral idiosyncrasies, with significance assessed using Mantel tests (10,000 permutations). The same approach was applied to the other indices as well.

#### Controlling analyses

First, to ascertain whether the AnnaK effect on the neural idiosyncrasy during mentalizing was specific to the MTN, we selected the physical pain network as a control, which has been shown in previous research to be functionally distinct from the MTN.^16^^,^^33^ We conducted parallel analyses on the physical pain network as the MTN, and also conducted a 3 (dyad group: HH vs. HL vs. LL) × 2 (network: MTN vs. Pain) analysis of variance (ANOVA) on the LME models for each neural index to assess whether the AnnaK effects were present exclusively in the MTN.

Second, to further validate our neural findings related to mentalizing, we segmented the movie into three parts based on content. The first segment (1–50 TRs) served as background, where participants established the story framework without specific mentalizing. The second (51–110 TRs) and third (111–151 TRs) segments focused on the mental states of a bird and a cloud, respectively. We calculated the inter-subject dissimilarity of global functional connectivity and strength centrality and compared these neural indices between HH, HL, and LL dyad groups for each segment. Additionally, treating PT as a continuous variable, we examined correlations between inter-subject dissimilarity and mean PT values for each segment, using LME models.

Lastly, to confirm that the AnnaK effects observed were specific to social information processing rather than general intrinsic neural activity, we conducted parallel analyses on the neural activity in the MTN during rest. We included the neural indices from the resting-state as covariates in the LME models to assess whether our previous results persisted. Furthermore, we examined the association between the inter-subject dissimilarity of verbal interpretation and that of neural indices (i.e., global functional connectivity and strength centrality) during rest, using Pearson correlations assessed with Mantel tests (*n* = 10,000 permutations).
