# Supplementary material for: Sophisticated perspective-takers are distinctive: Neural idiosyncrasy of functional connectivity in the mentalizing network
Source: iScience. 2024 Nov 23;27(12):111472. doi: 10.1016/j.isci.2024.111472 (PMC11667172; doi:10.1016/j.isci.2024.111472)
Supplement: Document S1. Figures S1, S2 and Tables S1–S13 [file mmc1.pdf]

iScience, Volume *27*

Supplemental information

Sophisticated perspective-takers are distinctive: Neural idiosyncrasy of functional connectivity in the mentalizing network

Yu Zhang, Chao Ma, Haiming Li, Leonardo Assumpção, and Yi Liu

Supplementary Figure

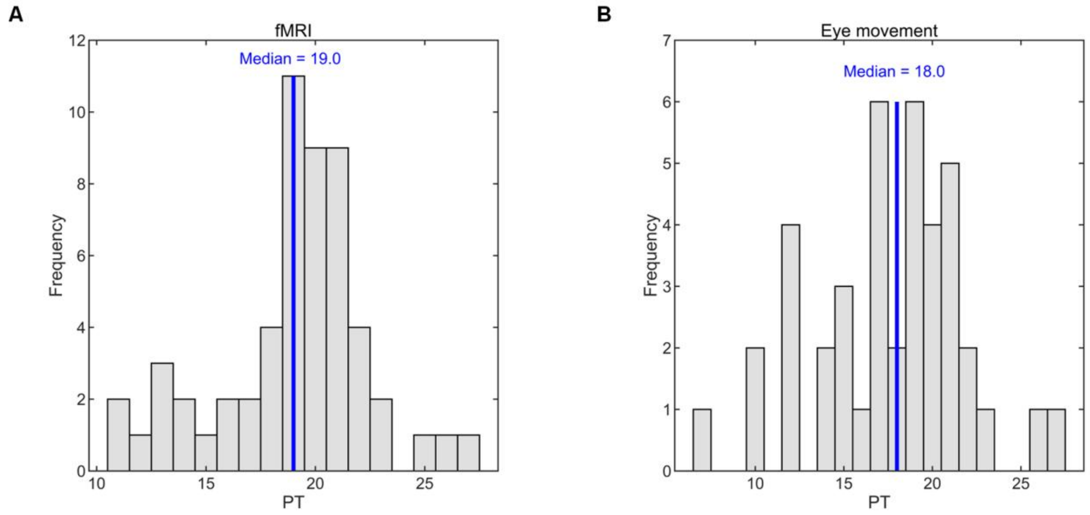

Figure S1. Distribution of perspective-taking (PT) scores.

(A and B) The distribution of PT scores for (A) the 55 participants in the fMRI experiment, and for (B) the 41 participants in the eye-movement experiment.

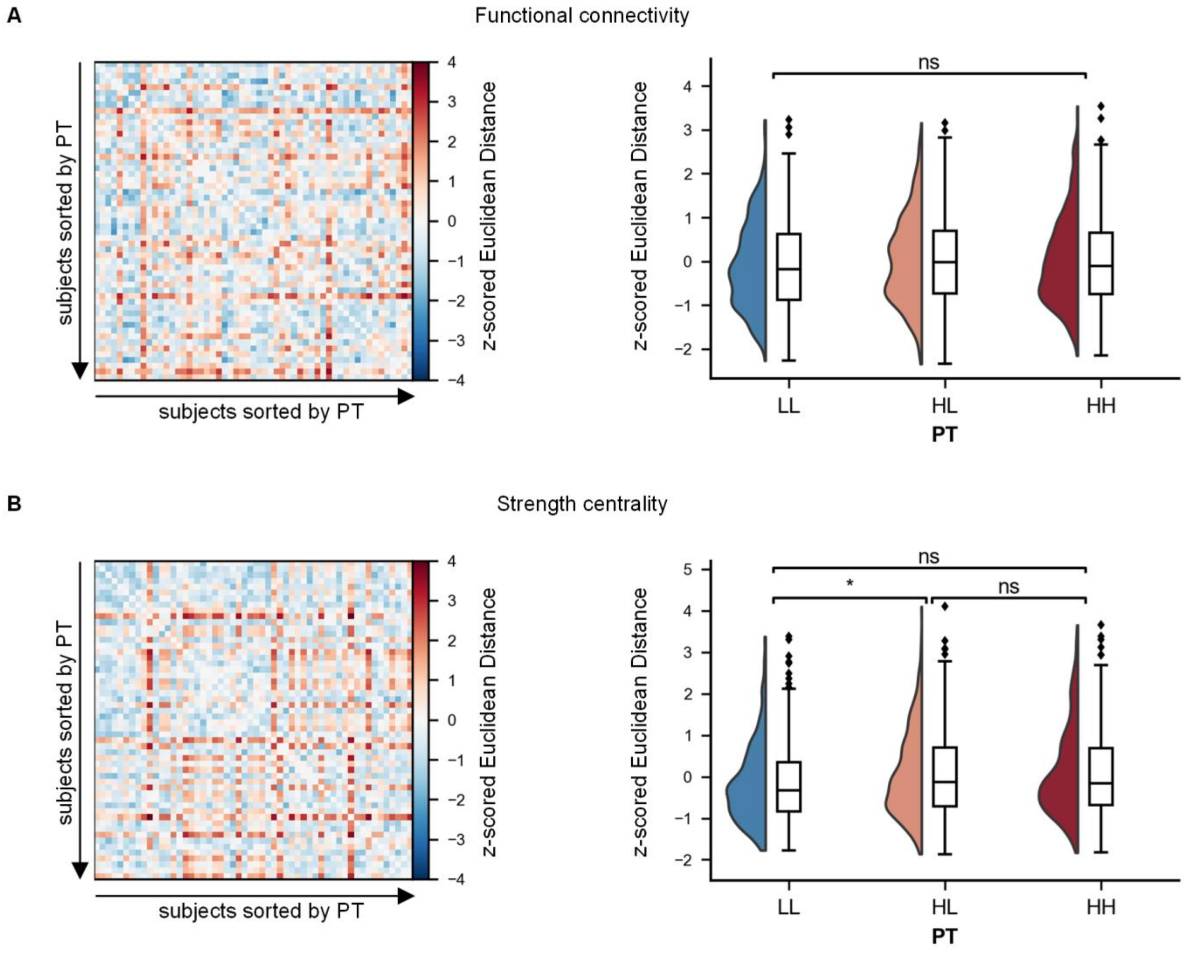

Figure S2. Inter-subject dissimilarity (i.e., Euclidean distance) of the functional connectivity and the strength centrality for the physical pain network.

(A and B) Left panel: inter-subject dissimilarity matrix of the functional connectivity and strength centrality within the pain network; the rows and columns of the matrix are ordered by increasing PT scores. The colors in the left panels stand for the standardized Euclidean distance of the global functional connectivity and strength centrality for each dyad. Right panel: mean inter-subject dissimilarity in three dyad groups (i.e., HH, HL, and LL). The half-violin plots represent the distribution of data. Boxes in the right panels represent the interquartile range; horizontal lines indicate the median and whiskers extend 1.5 times the interquartile range from the first and third quartiles. *$p < 0.05$.

Supplementary Tables

Table S1. The inter-subject correlation of mentalizing network during video watching and rest.

ROI	Video		Rest		*t*	*p*
	M	SE	M	SE		
RTPJ	0.133	0.017	0.001	0.013	32.427	< 0.001
LTPJ	0.083	0.015	0.001	0.013	20.894	< 0.001
PreC	0.175	0.021	0.000	0.014	36.531	< 0.001
DMPFC	0.051	0.014	0.004	0.013	12.708	< 0.001
MMPFC	0.050	0.014	0.005	0.012	12.749	< 0.001
VMPFC	0.021	0.013	0.002	0.012	5.240	< 0.001

Table S2. Comparisons of other variables between the high and low PT groups and Spearman correlation between PT scores and other variables.

Variable			Categorical (low vs. high)		Continuous	
			t	*p*	Spearman *r*	*p*
fMRI	Demographics	Age	0.915	0.364	0.012	0.933
		Sex[a]	0.439	0.508	–	–
	Interpersonal Reactivity Index	Fantasy	-1.532	0.131	0.339	**0.011**
		Empathy	-2.464	**0.017**	0.398	**0.003**
		Personal distress	-0.342	0.734	0.141	0.304
	Big-Five personality traits	Neuroticism	0.386	0.701	-0.101	0.462
		Extraversion	-0.414	0.681	0.178	0.193
		Openness	-1.028	0.309	0.123	0.370
		Agreeableness	0.694	0.491	0.046	0.739
		Conscientiousness	0.309	0.758	-0.037	0.789
Eye-movement	Demographics	Age	-0.384	0.703	-0.063	0.696
		Sex[a]	0.223	0.636	–	–
	Interpersonal Reactivity Index	Fantasy	0.489	0.628	-0.082	0.612
		Empathy	0.153	0.879	0.102	0.526
		Personal distress	1.058	0.296	-0.244	0.124
	Big-Five personality traits	Neuroticism	1.303	0.200	-0.348	**0.026**
		Extraversion	-0.903	0.372	0.311	**0.048**
		Openness	-0.620	0.539	0.212	0.183
		Agreeableness	-2.605	**0.013**	0.389	**0.012**
		Conscientiousness	-1.462	0.152	0.403	**0.009**
Verbal interpretation	Demographics	Age	1.117	0.267	0.000	0.997
		Sex[a]	0.229	0.632	–	–
	Interpersonal Reactivity Index	Fantasy	-0.977	0.331	0.157	0.136
		Empathy	-1.771	**0.080**	0.237	**0.024**
		Personal distress	0.056	0.956	0.003	0.977
	Big-Five personality traits	Neuroticism	1.341	0.183	-0.187	0.075
		Extraversion	-0.873	0.385	0.218	**0.038**
		Openness	-1.147	0.254	0.142	0.178
		Agreeableness	-1.312	0.193	0.218	**0.038**
		Conscientiousness	-0.811	0.419	0.188	0.075

[a]Measured with chi-squared test

Table S3. Comparisons between dyad groups on the inter-subject dissimilarity of time dynamics in the mentalizing network, when controlling for potential confounding variables.

ROI	Contrast	β	SE	*p* (uncorrected)	*p* (corrected)
VMPFC	HH - LL	-0.256	0.142	0.011	0.065
VMPFC	HH - HL	-0.216	0.087	< 0.001	0.008
VMPFC	HL - LL	-0.040	0.087	0.508	0.609
DMPFC	HH - LL	0.301	0.166	0.010	0.065
DMPFC	HH - HL	0.138	0.096	0.043	0.111
DMPFC	HL - LL	0.163	0.096	0.015	0.070
PreC	HH - LL	0.433	0.300	0.041	0.111
PeC	HH - HL	0.190	0.154	0.081	0.182
PreC	HL - LL	0.243	0.154	0.025	0.090
LTPJ	HH - LL	0.163	0.228	0.314	0.435
LTPJ	HH - HL	0.050	0.122	0.565	0.623
LTPJ	HL - LL	0.113	0.122	0.191	0.344
RTPJ	HH - LL	0.209	0.272	0.277	0.415
RTPJ	HH - HL	0.078	0.141	0.435	0.559
RTPJ	HL - LL	0.131	0.141	0.189	0.344
MMPFC	HH - LL	0.067	0.176	0.588	0.623
MMPFC	HH - HL	-0.021	0.100	0.772	0.772
MMPFC	HL - LL	0.088	0.100	0.212	0.346

Table S4. Correlation between mean PT scores and inter-subject dissimilarity of time dynamics for the mentalizing network, when controlling for potential confounding variables.

ROI	β	*p* (uncorrected)	*p* (corrected)
PreC	0.171	0.037	0.192
VMPFC	-0.072	0.064	0.192
LTPJ	0.072	0.234	0.360
MMPFC	0.052	0.269	0.360
DMPFC	0.047	0.300	0.360
RTPJ	0.033	0.655	0.655

Table S5. Comparisons between dyad groups on the inter-subject dissimilarity of functional connectivity in the mentalizing network, when controlling for potential confounding variables.

Functional connectivity	Contrast	β	SE	*p* (uncorrected)	*p* (corrected)
LTPJ - DMPFC	HH - LL	0.625	0.275	0.001	**0.030**
	HH - HL	0.300	0.143	0.003	**0.044**
	HL - LL	0.325	0.143	0.001	**0.030**
LTPJ - MMPFC	HH - LL	0.438	0.268	0.021	0.188
	HH - HL	0.189	0.140	0.055	0.261
	HL - LL	0.248	0.140	0.012	0.131
RTPJ - MMPFC	HH - LL	0.380	0.246	0.029	0.188
	HH - HL	0.176	0.130	0.056	0.261
	HL - LL	0.204	0.130	0.026	0.188
RTPJ - LTPJ	HH - LL	0.396	0.298	0.061	0.261
	HH - HL	0.200	0.154	0.066	0.261
	HL - LL	0.197	0.154	0.070	0.261
DMPFC - MMPFC	HH - LL	0.297	0.299	0.161	0.387
	HH - HL	0.156	0.153	0.151	0.387
	HL - LL	0.141	0.153	0.194	0.387
DMPFC - VMPFC	HH - LL	0.242	0.256	0.182	0.387
	HH - HL	0.150	0.135	0.114	0.387
	HL - LL	0.091	0.135	0.335	0.481
LTPJ - PreC	HH - LL	0.186	0.270	0.332	0.481
	HH - HL	0.121	0.141	0.223	0.387
	HL - LL	0.064	0.141	0.519	0.631
LTPJ - VMPFC	HH - LL	0.273	0.314	0.218	0.387
	HH - HL	0.136	0.161	0.232	0.387
	HL - LL	0.138	0.161	0.226	0.387
PreC - VMPFC	HH - LL	0.306	0.334	0.194	0.387
	HH - HL	0.152	0.170	0.204	0.387
	HL - LL	0.154	0.170	0.199	0.387
RTPJ - PreC	HH - LL	0.312	0.295	0.136	0.387
	HH - HL	0.157	0.152	0.145	0.387
	HL - LL	0.155	0.152	0.149	0.387
RTPJ - DMPFC	HH - LL	0.182	0.270	0.342	0.481
	HH - HL	0.112	0.141	0.262	0.407
	HL - LL	0.070	0.141	0.482	0.603
RTPJ - VMPFC	HH - LL	0.175	0.281	0.377	0.514
	HH - HL	0.119	0.146	0.248	0.398
	HL - LL	0.057	0.146	0.582	0.689
PreC - DMPFC	HH - LL	0.073	0.276	0.708	0.797
	HH - HL	0.077	0.144	0.446	0.574
	HL - LL	-0.004	0.144	0.966	0.988
PreC - MMPFC	HH - LL	0.083	0.300	0.695	0.797
	HH - HL	-0.009	0.154	0.935	0.979
	HL - LL	0.092	0.154	0.399	0.528
MMPFC - VMPFC	HH - LL	-0.025	0.284	0.900	0.964
	HH - HL	0.000	0.147	0.997	0.997
	HL - LL	-0.026	0.147	0.804	0.883

Table S6. Comparisons of mean functional connectivity between the high and low PT groups

Functional connectivity	Low PT		High PT		*t*	*p* (uncorrected)	*p* (corrected)
	M	SE	M	SE			
PreC - MMPFC	0.461	0.032	0.566	0.036	-2.200	0.032	0.483
LTPJ - MMPFC	0.579	0.033	0.649	0.043	-1.291	0.202	0.917
RTPJ - MMPFC	0.443	0.032	0.507	0.042	-1.222	0.227	0.917
PreC - VMPFC	0.260	0.036	0.322	0.045	-1.068	0.290	0.917
MMPFC - VMPFC	0.265	0.043	0.313	0.041	-0.812	0.420	0.917
LTPJ - VMPFC	0.242	0.039	0.287	0.044	-0.783	0.437	0.917
RTPJ - PreC	0.820	0.037	0.855	0.046	-0.588	0.559	0.917
DMPFC - VMPFC	0.205	0.031	0.234	0.039	-0.583	0.562	0.917
LTPJ - DMPFC	0.627	0.042	0.666	0.061	-0.522	0.604	0.917
RTPJ - DMPFC	0.449	0.041	0.418	0.045	0.511	0.611	0.917
RTPJ - VMPFC	0.229	0.037	0.249	0.038	-0.393	0.696	0.949
LTPJ - PreC	0.798	0.041	0.806	0.046	-0.124	0.902	0.966
PreC - DMPFC	0.518	0.032	0.513	0.034	0.095	0.924	0.966
DMPFC - MMPFC	0.719	0.053	0.723	0.053	-0.049	0.961	0.966
RTPJ - LTPJ	0.875	0.034	0.877	0.050	-0.043	0.966	0.966

Table S7. Comparisons between dyad groups on the inter-subject dissimilarity of strength centrality for the single regions in the mentalizing network, when controlling for potential confounding variables.

ROI	Contrast	β	SE	*p* (uncorrected)	*p* (corrected)
DMPFC	HH - LL	0.500	0.278	0.011	**0.078**
	HH - HL	0.266	0.145	0.009	**0.078**
	HL - LL	0.233	0.145	0.022	**0.078**
MMPFC	HH - LL	0.355	0.228	0.028	**0.078**
	HH - HL	0.169	0.123	0.052	**0.078**
	HL - LL	0.186	0.123	0.031	**0.078**
PreC	HH - LL	0.379	0.257	0.037	**0.078**
	HH - HL	0.177	0.135	0.063	**0.088**
	HL - LL	0.202	0.135	0.033	**0.078**
RTPJ	HH - LL	0.370	0.248	0.035	**0.078**
	HH - HL	0.182	0.132	0.051	**0.078**
	HL - LL	0.189	0.132	0.041	**0.078**
LTPJ	HH - LL	0.376	0.294	0.070	**0.090**
	HH - HL	0.166	0.152	0.123	0.147
	HL - LL	0.211	0.152	0.049	**0.078**
VMPFC	HH - LL	0.191	0.302	0.369	0.391
	HH - HL	0.120	0.155	0.277	0.311
	HL - LL	0.072	0.155	0.512	0.512

Table S8. Comparisons of strength centrality in single regions between the high and low PT groups

ROI	Low PT		High PT		*t*	*p* (uncorrected)	*p* (corrected)
	M	SE	M	SE			
MMPFC	2.467	0.120	2.701	0.155	-1.205	0.234	0.887
PreC	2.857	0.116	3.007	0.159	-0.767	0.446	0.887
VMPFC	1.200	0.163	1.328	0.180	-0.529	0.599	0.887
LTPJ	3.122	0.131	3.218	0.164	-0.465	0.644	0.887
RTPJ	2.815	0.124	2.862	0.169	-0.230	0.819	0.887
DMPFC	2.518	0.129	2.486	0.182	0.143	0.887	0.887

Table S9. The ROI definitions of the pain network (selected from ref. 16)

ROI	x MNI(mm)	y MNI(mm)	z MNI(mm)
R. secondary sensory	60	-28	38
L. secondary sensory	-62	-32	34
R. insula	42	6	-6
L. insula	-42	-2	-4
R. medial frontal gyrus	50	42	12
L. medial frontal gyrus	-46	36	14
Anterior middle cingulate cortex	0	2	42

Table S10. Comparisons between dyad groups on the inter-subject dissimilarity of global functional connectivity and strength centrality in the pain network, when controlling for potential confounding variables.

	Contrast	β	SE	*p* (uncorrected)	*p* (corrected)
Functional connectivity	HH - LL	0.213	0.301	0.316	0.474
	HH - HL	0.045	0.155	0.680	0.680
	HL - LL	0.168	0.154	0.123	0.370
Strength centrality	HH - LL	0.318	0.263	0.088	0.132
	HH - HL	0.072	0.138	0.460	0.460
	HL - LL	0.245	0.137	0.012	0.035

Table S11. Comparisons between dyad groups on the inter-subject dissimilarity of global functional connectivity and strength centrality for the mentalizing network in the three segments, when controlling for potential confounding variables.

	Segment	Contrast	β	SE	*p* (uncorrected)	*p* (corrected)
Functional connectivity	Segment1	HH - LL	0.109	0.320	0.630	0.708
		HH - HL	0.073	0.163	0.529	0.680
		HL - LL	0.036	0.163	0.752	0.752
	Segment2	HH - LL	0.596	0.286	0.003	**0.007**
		HH - HL	0.309	0.148	0.003	**0.007**
		HL - LL	0.287	0.148	0.006	**0.010**
	Segment3	HH - LL	0.575	0.264	0.002	**0.007**
		HH - HL	0.309	0.138	0.002	**0.007**
		HL - LL	0.265	0.137	0.006	**0.010**
Strength centrality	Segment1	HH - LL	0.207	0.294	0.320	0.339
		HH - HL	0.105	0.152	0.330	0.339
		HL - LL	0.102	0.152	0.339	0.339
	Segment2	HH - LL	0.539	0.261	0.004	**0.020**
		HH - HL	0.277	0.137	0.004	**0.020**
		HL - LL	0.262	0.136	0.007	**0.020**
	Segment3	HH - LL	0.402	0.236	0.016	**0.029**
		HH - HL	0.227	0.126	0.011	**0.025**
		HL - LL	0.175	0.125	0.047	**0.071**

Table S12. Comparisons between dyad groups on the inter-subject dissimilarity of global functional connectivity and strength centrality for the mentalizing network in the resting state, when controlling for potential confounding variables.

	Contrast	β	SE	*p* (uncorrected)	*p* (corrected)
Functional connectivity	HH - LL	0.226	0.287	0.266	0.335
	HH - HL	0.125	0.150	0.237	0.335
	HL - LL	0.101	0.148	0.335	0.335
Strength centrality	HH - LL	0.056	0.28	0.776	0.906
	HH - HL	0.044	0.146	0.669	0.906
	HL - LL	0.012	0.145	0.906	0.906

Table S13. Comparisons between dyad groups on the inter-subject dissimilarity of neural indices, when controlling for that of resting state and potential confounding variables.

	Contrast	β	SE	*p* (uncorrected)	*p* (corrected)
Functional connectivity	HH - LL	0.437	0.290	0.033	**0.050**
	HH - HL	0.235	0.150	0.027	**0.050**
	HL - LL	0.202	0.149	0.056	**0.056**
Strength centrality	HH - LL	0.515	0.256	0.004	**0.007**
	HH - HL	0.247	0.136	0.010	**0.010**
	HL - LL	0.267	0.133	0.005	**0.007**
